# To Christopher Dennett, Esq.

**Published:** 1803-08-01

**Authors:** John Ring

**Affiliations:** New Street, Hanover Square


To CHRISTOPHER DENNETT, Esq.

SIR,

I TRUST you will excuse my not answering your letter in this Journal for April, till I had time to pay that attention to it, which the importance of the subject demands. Accept my thanks for the flattering terms in which you speak of my endeavours to promote vaccination. It gives me sincere pleasure to find, that you, who have so often seen the inoculated Small-pox in its most favourable form, are become a strenuous advocate for the inoculation of the Cow-pock. No stronger argument of its decided superiority can be adduced.

I had

I had seen your letter in the Journal for September 1801; and have made some observations on it, which will appear in the second part of my treatise. The mode in which you preserve vaccine matter, is the same in which that was preserved, which was given to Mr. Cline by Dr. Jenner, in the year 1788; and which produced the first instance of vaccination in this metropolis. It is a mode, in which variolous matter had been preserved by Dr. Jenner, and other medical men.

Quills and ivory have been recommended, and employed, by different practitioners, in different forms. One gentleman, who has distinguished himself by his exertions in vaccination, uses the quill whole; and after charging the end of it well, inserts it into another; in the same manner as you direct. He also rubs it on the incision till the matter is dissolved.

Having been assured by Dr. De Carro, that his ivory lancets had proved very instrumental in propagating the practice, I endeavoured to get some made after the same pattern as one which he sent me. Being disappointed by the person to whom I applied, I had recourse to a quill, as one of the best substitutes; knowing that it had often been used for the purpose of preserving matter, but not recollecting that it had been mentioned by you.

In one circumstance, however, and that, in my opinion, not a trifling one, the quill sharpened like a toothpick differs from that recommended by you; which is, that it admits of our performing the operation by a mere puncture. When performed by an incision, or as you recommend, by three or four incisions, it must be allowed, the chance of exciting the disease is still greater; but it is so much more violent, when excited, that I prefer the method of inoculating by puncture.

The probability of success, from using the matter on a toothpick, is great; and by its being divided into so many parts, the chance is multiplied. Dr. Waterhouse successfully inoculated six persons with two toothpicks, when the matter was two months old. He did not entertain any apprehension of danger to the patient, or offence to the parent, from vaccinating one person after another with the same instrument; such as Mr. Galliard alludes to in the last number. If there be any parents who harbour that foolish prejudice, it is the duty of medical men rather to remove it, than to root it in their minds.

It is well known, that the blood is not infectious. Were it otherwise, there would be danger of communicating all the the diseases of the person inoculated, to the person from whom matter is taken; when it is taken a second time, either with the intention of saturating the first puncture, or inoculating in the other arm, without washing the lancet every time. It is well known, that scarcely any inoculator is very scrupulous in this respect; but the oftener the lancet is washed the better, on another account; which is, that when it is less clogged with matter, it is less likely to produce a spurious pustule.

In a considerable number of instances, two or three persons have been inoculated with one lancet of dry matter, which I had sent to medical gentlemen, and others, in various parts of the kingdom; and surely this was not only excuseable, but commendable; since, in a great proportion of those cases, the small-pox was either in the same place, or in the neighbourhood.

It is in consequence of this necessity of exciting the guardian disease with all possible expedition; a necessity, alas! which too frequently occurs, and too frequently will occur, till medical practitioners are more provident, and more careful, either to maintain a succession of cow-pock patients, or to preserve a stock of vaccine matter, that I thought it necessary to mention, that more than one patient might be inoculated with one vaccinator.

At present, when the practice of vaccination prevails in almost every town, those who want cow-pock matter may, perhaps, be accommodated as they wish. It was far otherwise when there were few sources of supply; and still fewer in which the public could place much confidence.

In a former number of this Journal, an apprehension was expressed by Dr. Marcet, that the influence of some persons might occasion a discontinuance of the use of glass, as a preserver of vaccine virus. If his partiality in favour of that method were well founded, it would not readily be laid aside; but it is found so frequently to fail, that those gentlemen by whose influence it was established, now candidly confess, it has not answered their expectations.

This agrees with the general testimony of the most distinguished inoculators in other parts of the world; particularly, Dr. Waterhouse and Dr. De Carro. It is therefore, of some importance that it should be known; on account of the difficulty of collecting such quantities of matter, the injury done to the arms of those under vaccination by collecting it, and the disappointment which attends the want of success.

Your case of cow-pock, in a subject who appeared totally unsusceptible of the small-pox, is extremely interesting. Nevertheless it is not impossible, that at some future period a change in the constitution might have taken place. A parallel instance of this sort I have recorded: it was communicated to me by Dr. Bancroft, jun. His brother was inoculated for the small-pox eleven times; the last time by Dr. James Sims, in the Small-pox Hospital, in both arms, with recent variolous matter; but in vain. He afterwards caught the disease in the natural way, and had it severely.

A child of Mrs. Strachan, at No 48, Carnaby Street, died of the small-pox. Another child, who lay in the same bed, resisted infection. Since that time, I inoculated it with vaccine matter, which readily succeeded. These cases may have some effect in guarding medical practitioners against a hasty conclusion, that any one is insusceptible of variolous or vaccine infection.

New Street, Hanover Square, I am, &c.

July 18, 1803. JOHN RING.

